# High Recognition of Isomer-Stabilized Gold Nanoparticles through Matrix Imprinting

**DOI:** 10.1021/acsami.3c04311

**Published:** 2023-06-26

**Authors:** Din Zelikovich, Pavel Savchenko, Daniel Mandler

**Affiliations:** Institute of Chemistry, The Hebrew University of Jerusalem, Jerusalem 9190401, Israel

**Keywords:** gold nanoparticles, imprinting, nanoparticles detection, nanoparticles imprinted matrices (NAIM), Raman spectroscopy

## Abstract

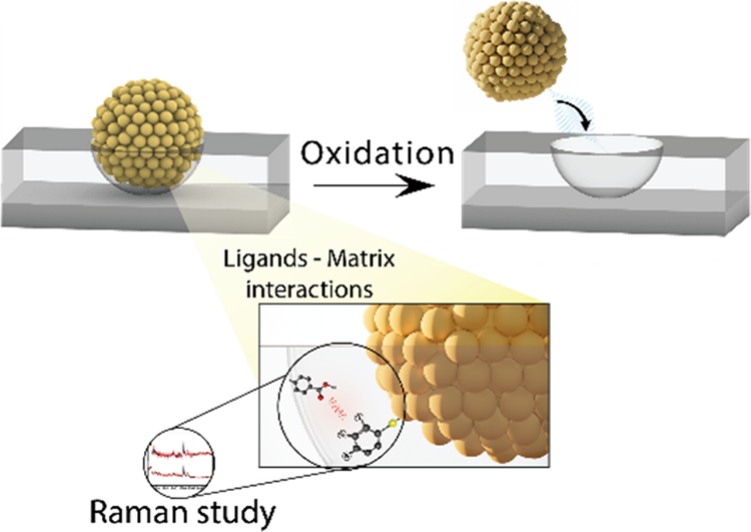

The development of highly selective probes for nanoparticles is required due to their nanotoxicity. The latter strongly depends on the size, structure, and interfacial properties of the nanoparticles. Here, we demonstrate that a simple approach for the selective detection of Au nanoparticles that differ in their capping agent shows very high promise. Specifically, gold nanoparticles stabilized by each of the three different isomers of mercaptobenzoic acid (MBA) were imprinted in a soft matrix by adsorption of the nanoparticles, followed by filling the non-occupied areas through electropolyermization of an aryl diazonium salt (ADS). Nanocavities bearing the shape of the Au nanoparticles were formed upon the electrochemical dissolution of the nanoparticles, which were used for the reuptake of the Au nanoparticles stabilized by the different isomers. High reuptake selectivity was found where the originally imprinted nanoparticles were recognized better than the Au nanoparticles stabilized by other MBA isomers. Furthermore, an imprinted matrix by nanoparticles stabilized by 4-MBA could also recognize nanoparticles stabilized by 2-MBA, and vice versa. A detailed study using Raman spectroscopy and electrochemistry disclosed the organization of the capping isomers on the nanoparticles as well as the specific nanoparticle-matrix interactions that were responsible for the high reuptake selectivity observed. Specifically, the Raman band at ca. 910 cm^–1^ for all AuNP–matrix systems implies the formation of a carboxylic acid dimer and thus the interaction of the ligands with the matrix. These results have implications for the selective and simple sensing of engineered nanoparticles.

## Introduction

Interactions of nanoparticles (NPs) with a matrix,^1^ polymer,^2^ or membrane play a critical role in various fields such as polymer nanocomposites and NP permeation across cell membranes.^3,4^ For example, interactions between NPs and polymers strongly affect the properties of nanocomposites where strong interactions result in uniform and superior materials^5^ such as an increase in the glass transition temperature. Furthermore, nanomaterials have been widely used in many biomedical applications,^6^ for example, cellular imaging,^7^ drug delivery,^8^ and cancer treatment,^9^ which require precise control over the NP–cell interaction. For these applications, NPs have to overcome the cell membrane barrier through either spontaneous penetration, or endocytosis, which makes their initial interaction with the membrane crucial.^10^ It is well documented that the size, shape, and charge of the NPs as well as their surface functionality strongly influence their penetration through the membrane.^11^

Special attention has been paid during the last years to gold NPs (AuNPs) and their interaction with cells.^12^ The unique qualities of AuNPs such as their low toxicity, biocompatibility, and tunable surface functionality have enabled a wide range of NP–cell interaction studies.^13^ These have shown that the surface charge as well as the shape and stabilizing shell of AuNPs lead to a different level of cellular uptake.

Many techniques are nowadays used for determining the physical properties of NPs such as transmission electron microscopy (TEM), dynamic light scattering, and more.^14^ However, these techniques provide limited information about the NP–matrix interaction. The latter has been studied by, for example, confocal laser scanning microscopy using fluorescent-labeled NPs as probes,^15^ enabling the simultaneous imaging of the cell and the NPs. Other light-scattering techniques such as dark field microscopy and flow cytometry have been applied to quantify and determine NP behavior within cells.^16^ The interactions of NPs embedded in nanocomposites are very often studied by IR spectroscopy, which can identify and characterize hydrogen and covalent bonding.

A molecularly imprinted polymer (MIP) is an established and well-known approach for sensing molecules.^17−19^ MIP formation is based on the polymerization of monomers in the presence of the template molecules; as a result, the template molecules integrate inside the polymeric matrix. Finally, the recognition sites (cavities) are created by the extraction of the template molecules. The recognition ability of these cavities is related to the size, shape, functional groups, chirality, and so forth of the template molecule.^20^

Recently, the MIP concept has been expanded for the imprinting of large entities such as NPs, viruses, and cells.^21^ In this emerging field termed surface-imprinted polymers (SIPs), a thin matrix imprints only part of the entity to enable its easy removal and rebinding. In the SIP approach, imprinting is achieved by the formation of a thin layer, thinner than the template dimension, on a solid support.^22^

Recently, we introduced a different approach inspired by the MIP approach, termed NP-imprinted matrices (NAIM) that enables targeting the NP–matrix interactions. In the NAIM approach, nanocavities with specific sizes, shapes, and chemical compositions are formed through the removal of imprinted NPs in thin matrices. These cavities are used to reuptake NPs very selectively based on their size, shape, and surface properties. The selectivity originates from both physical, that is, size and shape of the NP, as well as chemical matching between the NPs and the nanocavities. We have shown that such chemical and physical matching made it possible to differentiate, on the one hand, between AuNPs of different sizes^23^ and, on the other hand, between NPs stabilized by different capping agents.^1^ Whereas the NAIM concept has resulted in an incredible selectivity, which is partially due to NP–matrix interactions, it does not disclose the physicochemical nature of these interactions that should be investigated by spectroscopy. Surface-enhanced Raman spectroscopy (SERS) is a prominent optical tool for investigating the interactions, such as the adsorption of molecules and polymers on metallic nanostructures (mostly silver and gold) due to the strong localized surface plasmon resonance.^24^ For this reason, imprinted AuNPs in functionalized matrices are ideal Raman active “hot spots” expected to amplify the NP–matrix interaction signals.^25^

**Figure 1:**
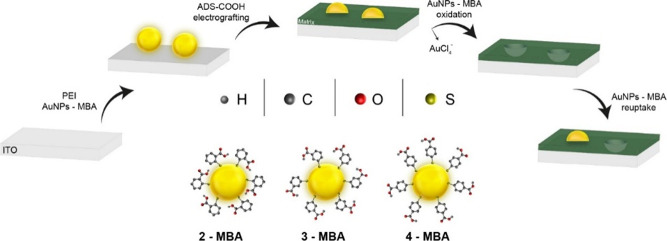
Schematics of the NAIM approach: ITO treated with PEI followed by the adsorption of AuNPs stabilized by the three MBA isomers. ADS-COOH was electrografted on the ITO to form the matrix, and the AuNPs were removed by electrochemical oxidation to form nanocavities, which were used to reuptake the originally imprinted AuNPs.

Here, we describe a NAIM-Raman combined study where we carefully examined the imprinting and recognition of AuNPs stabilized by the three isomers of mercaptobenzoic acid (MBA) in an aryldiazonium electropolymerized based matrix. Specifically, identical 10 nm diameter AuNPs stabilized by the 2, 3, and 4-MBA isomers were formed by a ligand-exchange reaction. Their adsorption on an indium tin oxide (ITO) surface modified by a positively charged polymer, for example, polyethylenimine (PEI), was followed by the controlled electrografting of a thin 4-carboxyphenyl diazonium (ADS-COOH) film, as shown schematically in Figure 1. The AuNPs were electrochemically dissolved and the reuptake of the different isomer-stabilized AuNPs was studied by electrochemistry, Raman spectroscopy, and other techniques. We found a remarkable selectivity that must be attributed to chemical pairing, namely, to the specific interactions between the stabilizing isomer of the NP and the matrix. Specifically, the highest reuptake percentage, that is, ranging from 60 to 80%, was found for the reuptake of the originally imprinted AuNPs, whereas the recognition of AuNPs bearing different MBA capping agents was substantially lower. The interactions between the MBA stabilizing the AuNPs and the aryldiazonium matrix were thoroughly studied by Raman spectroscopy and provided a molecular-level explanation for the performance of these NAIM systems.

## Results and Discussion

To examine the so-called “isomeric recognition effect” through the interaction between AuNPs and the matrix, we synthesized functionalized AuNPs stabilized by the three isomers of MBA (Figure 1). We anticipated that changes in the position of the carboxylic group in the MBA will likely affect the supramolecular NP–matrix interactions, which will be expressed in the recognition of the AuNPs by the matrix.

Imprinting of nanometric size entities, such as NPs, requires that the particles will have narrow-size distribution. Therefore, the AuNPs were grown following the Turkevich synthesis,^26^ where the seed stage ceased at ca. 8–10 nm. The initial citrate synthesized AuNPs were ligand-exchanged by ortho, meta, and para isomers of MBA. The challenge was to preserve the uniform and narrow-size distribution of the AuNPs while exchanging the ligands. Hence, the conditions of the ligand-exchange process were attentively maintained (see the Experimental Section). Typical TEM images of the AuNPs stabilized with 2-MBA are shown in Figure S1. Figure S2 shows the size distribution histogram and Gaussian fitting after exchanging the citrate with the MBA isomers. It is noticeable that the size and the spherical shape are retained.

The stability of the AuNPs-MBA was examined by measuring the ζ-potential, which is listed in Table S1. It can be seen that all ζ-potentials are negative, which is essential for stabilizing the AuNP dispersions. Exchanging the ligand from citrate (trivalent)^27^ to MBA ligands (mono- or divalent) decreases somewhat the surface potential, which might increase the tendency of the NPs to aggregate.^28^ The p*K*_HA_ of the ligands in solution is p*K*_cit_ = 3.13, 4.76, and 6.40,^29^ p*K*_2-MBA_ = 3.50, p*K*_3-MBA_ = 3.95, and p*K*_4-MBA_ = 4.8, and therefore, it is expected that the ζ-potential will be negative at pH > 5 in all cases. The ζ-potentials are in good agreement with the reported values.^1,30^ Yet, it should be noted that the p*K*_HA_ are those reported in solution, and there might be some differences for these monolayers on a surface. This issue was studied by Szleifer and co-workers,^31^ who concluded that the apparent p*K*a of the NP stabilized by capping ligands lies between that of free ligands and ligands self-assembled on a flat surface. This, alongside the careful cleaning of the AuNPs (see the Experimental Section), indicates that the ligand exchange was successful, while maintaining the shape and size of the AuNPs.

The UV–vis spectra of the different AuNPs are shown in Figure S3. The λ_max_ of the citrate, 2-MBA, 3-MBA, and 4-MBA stabilized AuNPs are 521, 523, 524, and 523 nm, respectively. According to the Mie-Gans model, spherical-shaped gold clusters have a unique surface plasmon resonance peak at ca. 520 nm.^32^ The single absorbance at ca. 524 nm and the absence of a peak at 650 nm for all the AuNPs imply that aggregation is negligible.^33^ The peak at the visible region and the red-shift of approximately 3 nm of the MBA-functionalized AuNPs (as compared with the AuNPs-cit) are due to the change in the dielectric environment at the AuNPs surface and also indicate an effective ligand exchange.^34^

The next step involved the adsorption of the NPs on an ITO electrode surface. As the AuNPs are negatively charged, it is conceivable to treat the ITO with a positively charged polymer, for example, PEI, and thus, adsorb the AuNPs through ionic interactions. Yet, it is essential to adsorb the particles evenly and discretely to create isolated imprinting sites. The ITO surfaces were treated with PEI solution for 2 h followed by washing with clean water for 24 h. This was crucial to dissolve long PEI chains partially linked to the ITO surface, which caused aggregation of the AuNPs in the solution and on the surface.

The ITO/PEI surfaces were submerged in the AuNPs-MBA solution for 1 h, which was found to be the optimal duration after which adsorption reached saturation (Figure S4). The amount of AuNPs adsorbed on the ITO/PEI surface was readily determined by linear sweep voltammetry (LSV) in HCl 0.1 M solution (Figure S4 A). The area under the LSV oxidation peak represents the charge associated with the oxidation of the AuNPs and therefore can be used as a sensitive probe for measuring the amount of adsorbed as well as reuptaken AuNPs.^1,35^Figure S5 proves that PEI is electrochemically inactive in the potential window of AuNP oxidation, which confirms that the peak area correlates exclusively with the AuNP oxidation charge. This identification approach will be used as the main analytical tool for the recognition of the NPs by the matrix.

**Figure 2:**
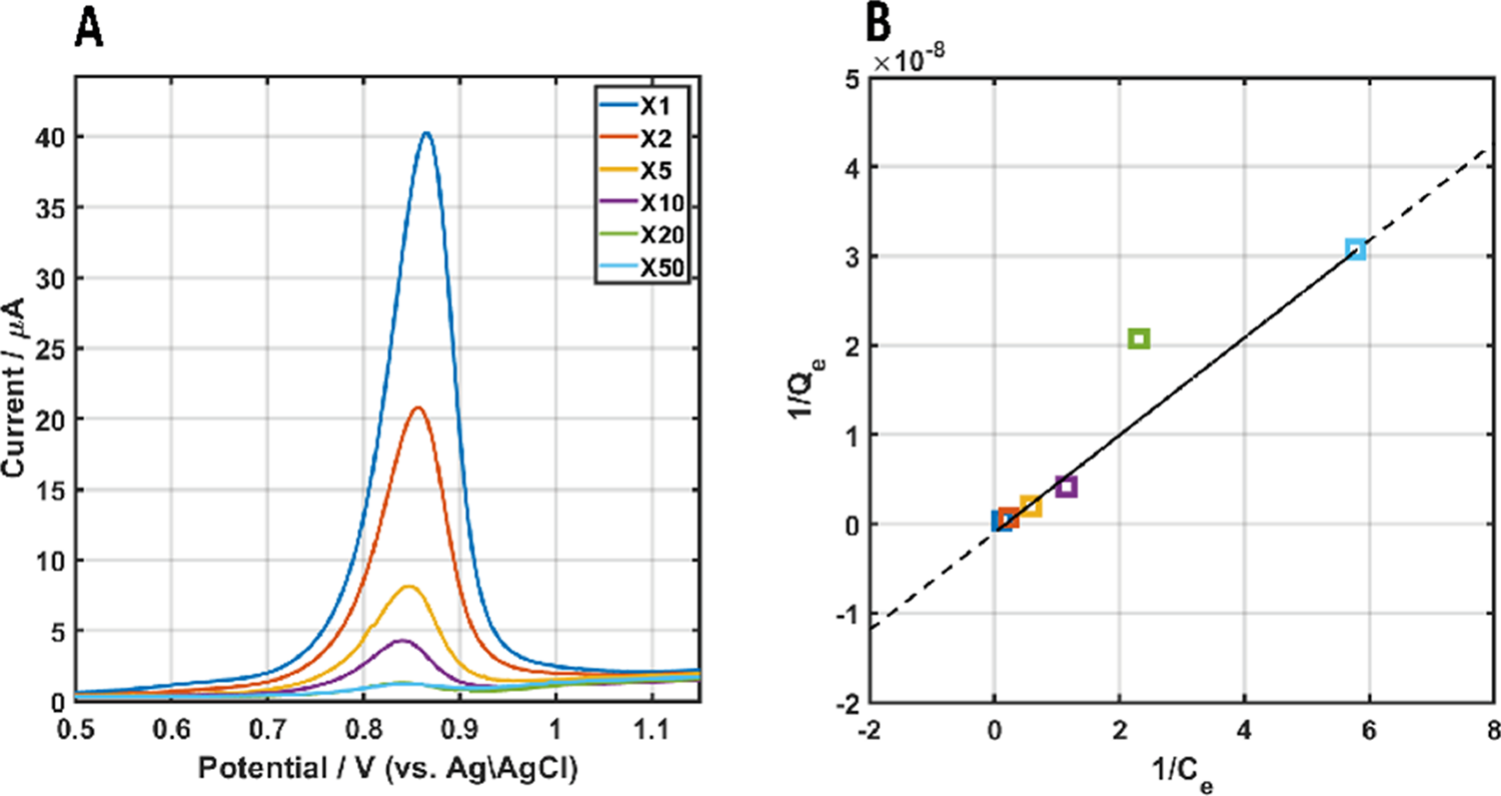
Measuring and analyzing the adsorption of AuNPs on ITO modified by PEI. (A) LSV of AuNPs-2-MBA for different NP concentrations in the adsorption solution (1 h of adsorption). (xn) represents the dilution factor of the original AuNP solution (see the Experimental Section), which is ca.  (B) Langmuir isotherm based on the charge shown in (A).

Figure 2A shows the dependence of the LSV for different adsorbing concentrations of AuNPs-2-MBA (adsorption time equals 1 h), which shows that the oxidation charge increases with the concentration of the AuNPs. Analyzing these data (Figure 2B) is based on the Langmuir isotherm, which is used to describe the adsorption of a monolayer that is independent of the coverage and expressed as follows:^36^
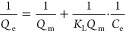
1where *Q*_e_ is the amount of adsorbed AuNPs (number of particles per area), *Q*_m_ is the maximum adsorption capacity for a monolayer coverage, *K*_L_ is the Langmuir adsorption constant that is related to the heat of adsorption, and *C*_e_ is the NPs concentration in the solution. The “separation factor” of the Langmuir isotherm, *R*_L_, is a measure of how favorable the adsorption process is and is described by:
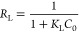
2where *C*_0_ is the initial AuNP concentration in mg L^–1^. *R*_L_ > 1 is an indicator of unfavorable adsorption, while 0 < *R*_L_ < 1 indicates favorable adsorption. When *R*_L_ = 1, termed linear process, there is no driving force for adsorption, whereas when *R*_L_ = 0, the adsorption is irreversible.

As can be seen in Figure 2B, the adsorption of AuNPs on an ITO/PEI is well described by the Langmuir isotherm model, which is in accordance with a previous report.^37^ The *R*_L_ for the Langmuir isotherm is 0.85 (Table 1), which indicates favorable adsorption and is supported by the scanning electron microscopy (SEM) images (Figure S6).

**Table 1:** Langmuir Parameters from the Fitted Models on the AuNP Adsorption Data

*Q*_m_ [NPs cm–2]	*K*_L_ [L mg^–1^]	*R*_L_	*R*^2^
1.13 × 10^10^	0.02	0.8523	0.9154

*Q*_m_, which is the saturated surface concentration of the AuNPs, is 1.13 × 10^10^ NPs cm^–2^, which is lower than that calculated from the SEM images (1.12 × 10^11^ NPs cm^–2^). We attribute this difference to the fact that the SEM images always represent a localized distribution of AuNPs. We believe that much of the area, especially in the periphery, has a lower AuNP density than observed by electron microscopy. Thus, we conclude that the adsorption is a consequence of the electrostatic interactions between the positively and negatively charged PEI and the AuNPs, respectively.

One of the most crucial steps in the NAIM system is to carefully form the matrix around the NPs. The importance of choosing an ADS matrix, as well as its advantages over other matrices, were shown in our previous work.^1^ Owing to the benefits of the ADS matrix as compared with a wide variety of other matrices, we synthesized ADS bearing a carboxylic acid following a known procedure (see the Experimental Section). Figure S7 shows the Raman spectrum of 4-carboxyphenyldiazonium (ADS-COOH) powder. A comparison between the observed band assignments of the ADS-COOH Raman spectrum and that calculated by DFT is given in Table S2 and shows significant similarities between the computed and observed values. Specifically, the strong band at 2308 cm^–1^, which confirms the presence of a N ≡ N bond, and the band at 1124 cm^–1^, which corresponds to C–N_2_ stretching, agree with the DFT model and are a specific fingerprint of the diazonium moiety.^38,39^ The most significant signature of the benzene ring is its ortho-meta C=C stretching mode recorded at 1073 and 1590 cm^–1^ which is the so-called “quinoid” vibration.^38,39^ In addition, the bands at 486 cm^–1^ and 1707–1732 cm^–1^ confirm the existence of the carboxylic acid functional group and are related to its deformation and C=O stretching, respectively.^40^ A video of the most significant vibrations of the diazonium molecule is shown in Figure S8.

Electrografting by cyclic voltammetry (CV) of ADS-COOH on the ITO/PEI/AuNPs modified surface is found to be optimal for three repetitive scans at a scan rate of 0.1 V s^–1^. The formation of the electrografted matrix on the ITO surface is characterized by an irreversible cathodic peak at ca. −0.23 V vs Ag/AgBr^1^. The evidence of the deposition of a blocking layer made of the electrografted ADS-COOH can be seen in Figure S9, where the CV of hexacyanoferrate(III) was studied as a function of the number of the electrografted CV cycles. It is evident that the CV current decreases with the increasing number of electrografted scans, which corresponds to the increasing film thickness.^22^ Specifically, three scans of ADS-COOH block electron transfer at an ITO/PEI/ADS-COOH surface without NPs, whereas the adsorption of the NPs clearly allows electron transfer. We have previously shown that electron transfer takes place at the imprinted NPs, providing that they are not fully covered. Hence, we can conclude that three deposition scans form a layer, which does not fully cover the NPs and yet prevent electron transfer in the areas that are not occupied by the NPs.

**Figure 3:**
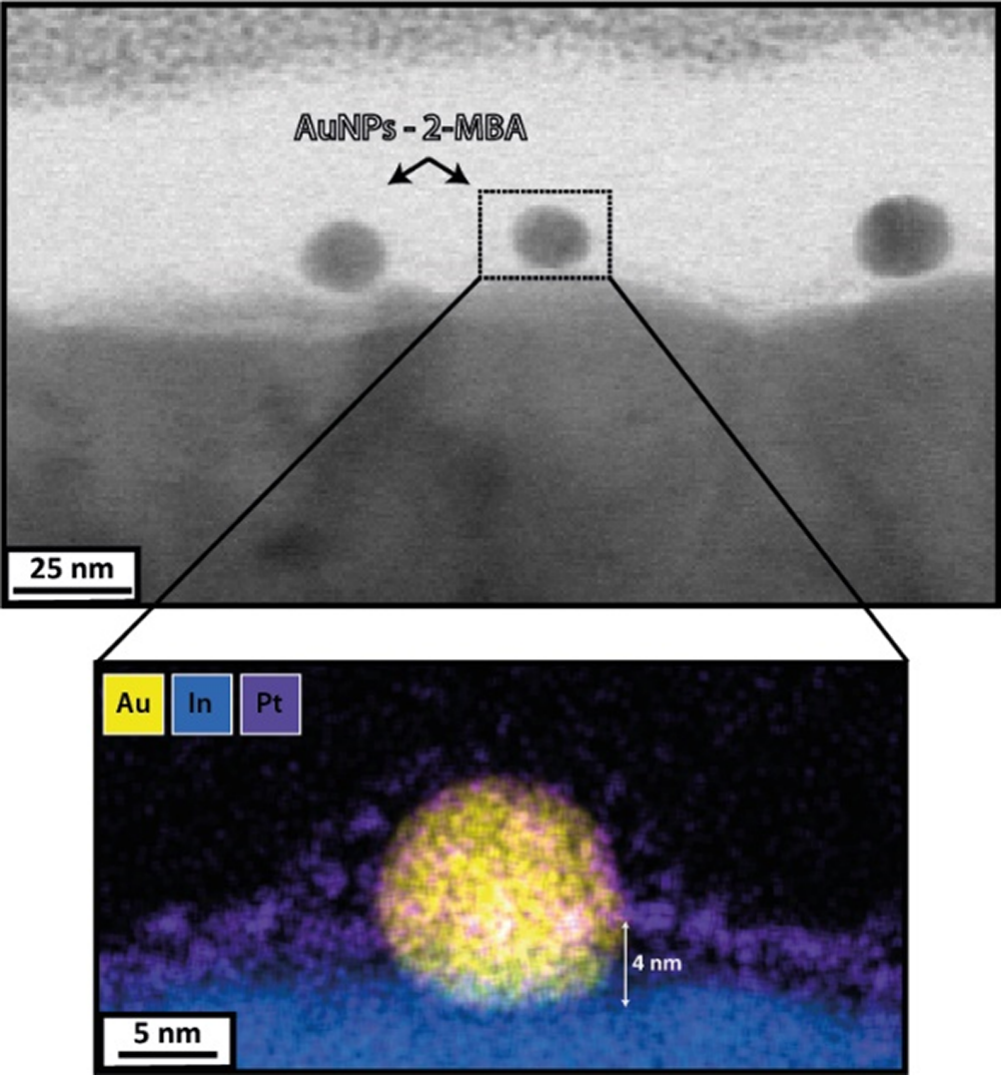
TEM image of focus ion beam (FIB) cross-section and EDS mapping of AuNPs-2-MBA embedded in a ADS-COOH layer electrografted by three CV scans.

The presence of carboxylic acids in the matrix promotes strong interaction between the imprinted AuNPs capping agents and the matrix.^1^ To study the imprinted AuNPs, a thin lamella of ITO/PEI/AuNPs-2-MBA electrografted with three scans of ADS-COOH was sliced and imaged by high-resolution cross-section TEM (Figure 3). It is noticeable that the imprinted AuNPs-2-MBA adsorbed on the functionalized ITO surface are ca. 10 nm and are partially covered by the ADS organic layer that is 4.1 ± 0.3 nm thick. We have previously shown that the optimal thickness of the NAIM layer should equal the radius of the NPs to allow their efficient removal by electrochemical dissolution.^41^ The SEM image (Figure S10) of the AuNPs-MBA on an ITO/PEI surface after forming the matrix indicates that the NPs are intact by electrografting.

The successful grafting of the diazonium salt on the ITO surface was confirmed by comparing the Raman spectrum of the synthesized ADS-COOH powder with the SERS spectra of the electrografted films (Tables S3–S5). The main evidence is the absence, in all three NAIM systems, of the most pronounced fingerprint of the diazonium moiety. Hence, the strong peak of N ≡ N stretching at 2308 cm^–1^ and C–N_2_ stretch at 1124 cm^–1^ are absent due to the electrochemical reduction of ADS-COOH on the electrode, which involves N_2_ cleavage and the formation of an aryl radical that reacts with the surface.^1,42^

Removal of the imprinted AuNPs-MBA was accomplished by scanning the surfaces from 0.5 to 1.2 V in 0.1 M HCl, which is the potential window of Au oxidation^43,44^ (Figure S11). The surface was scanned 10 times until the oxidation wave of AuNPs fully disappeared. This ensured the full dissolution of the AuNPs-MBA and the formation of nanovoids. SEM images (not shown) did not disclose any NPs on the surfaces after scanning the potential. Figure S11 shows that the first oxidation peak is significantly larger than the subsequent peaks and it provides a measure of the amount of charge of the AuNPs-MBA that are dissolved from the surface. It is worth noticing a small but constant negative shift of the oxidation peak upon repetitive scanning, which implies more facile oxidation of the NPs. This shift can be attributed to a decrease in the remaining Au core size with oxidation, which is supported by previous studies.^45,46^

The oxidation of the AuNPs-MBA is expected to leave nanocavities that should selectively recognize the originally imprinted NPs.^47^ Therefore, the next step involved the reuptake process where the surface after oxidation, that is, ITO\PEI\ADS-COOH, was immersed into the different AuNPs-MBA isomer solutions. This was followed by another oxidation scan that enabled determining the charge and therefore the number of NPs that were recognized by the NAIM. Figure S12 shows the effect of the immersion time on the reuptake percentage. The latter is defined as the ratio between the charge associated with the reuptake and the original oxidation. Evidently, reuptake reaches a plateau after ca. 1 h, and therefore, this time was selected as a standard for reuptake experiments.

The next step focused on probably the most interesting aspect, which is the selectivity of the imprinted systems toward different isomers. Comparison is based on the reuptake percentage, obtained by comparing the oxidation waves of the reuptake of a specific isomer-based AuNPs and that of the originally imprinted NPs. Specifically, this comparison is achieved by an automated method of analyzing the data after isolating the peak and fitting an appropriate baseline (see the Experimental Section). In this automated approach, a single standard is established to calculate the area under the peak.

**Figure 4:**
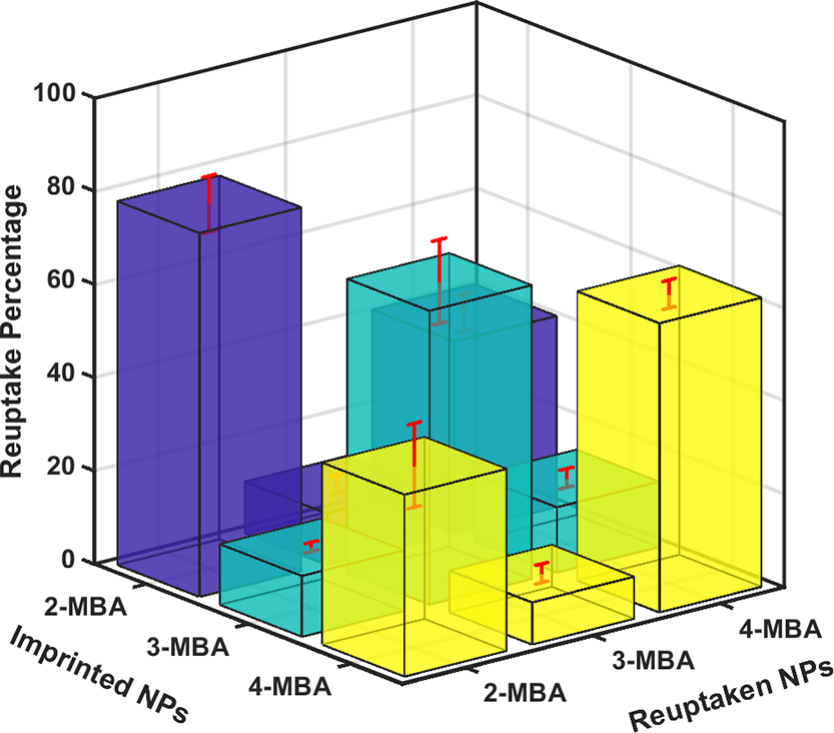
Reuptake percentage of the NAIM systems for the different MBA stabilized AuNPs (raw data are shown in Figure S13).

Figure 4 summarizes the reuptake percentage of nine different NAIM systems that are defined by the capping agents of the imprinted and reuptaken NPs. The LSV curves of these systems are shown in Figure S13. Furthermore, control experiments using non-imprinted matrices were carried out for all cases (as described in the Experimental Section); however, only one example (Figure S13 A-1 yellow curve) is shown. In all cases, non-specific adsorption is negligible, as no oxidation waves were detected. Moreover, the reuptake experiments were highly reproducible and repeated more than 10 times with an error smaller than 10%.

The highest reuptake percentage is observed for the NAIM systems recognizing the originally imprinted AuNPs-MBA. Specifically, the reuptake percentage of the imprinted 2-MBA, 3-MBA, and 4-MBA was 78, 63, and 62% for reuptaking the corresponding isomer-based AuNPs, respectively (see Figure S13 A-1, B-2, and C-3). Furthermore, the matrix imprinted by AuNPs-3-MBA (yellow columns) shows high selectivity, that is, the reuptake percentage of the other isomers-based AuNPs is low. On the other hand, both matrices imprinted by the 2- (blue columns) and 4-MBA (green column) AuNPs almost do not reuptake the AuNPs-3-MBA; however, the differentiation between the two other isomers, that is, AuNPs-2-MBA and AuNPs-4-MBA is low. Specifically, the imprinted AuNPs-2-MBA–matrix reuptakes the original AuNPs only 1.9 times more than it reuptakes the AuNPs-4-MBA, while the imprinted 4-MBA AuNPs reuptakes the originally imprinted AuNPs 1.6 times more than it recognizes the AuNPs-2-MBA. It is worth mentioning that the reproducibility of these measurements is high and the data presented are an average of numerous repetitive experiments.

The selectivity obtained by these systems is strikingly high and must be contributed to both the organization of the isomers on the AuNPs as well their interaction with the matrix, vide-infra. It should be emphasized that the selectivity cannot be attributed to differences in the size of the AuNPs since the same Au core was used for preparing the different isomer-stabilized NPs.

**Figure 5:**
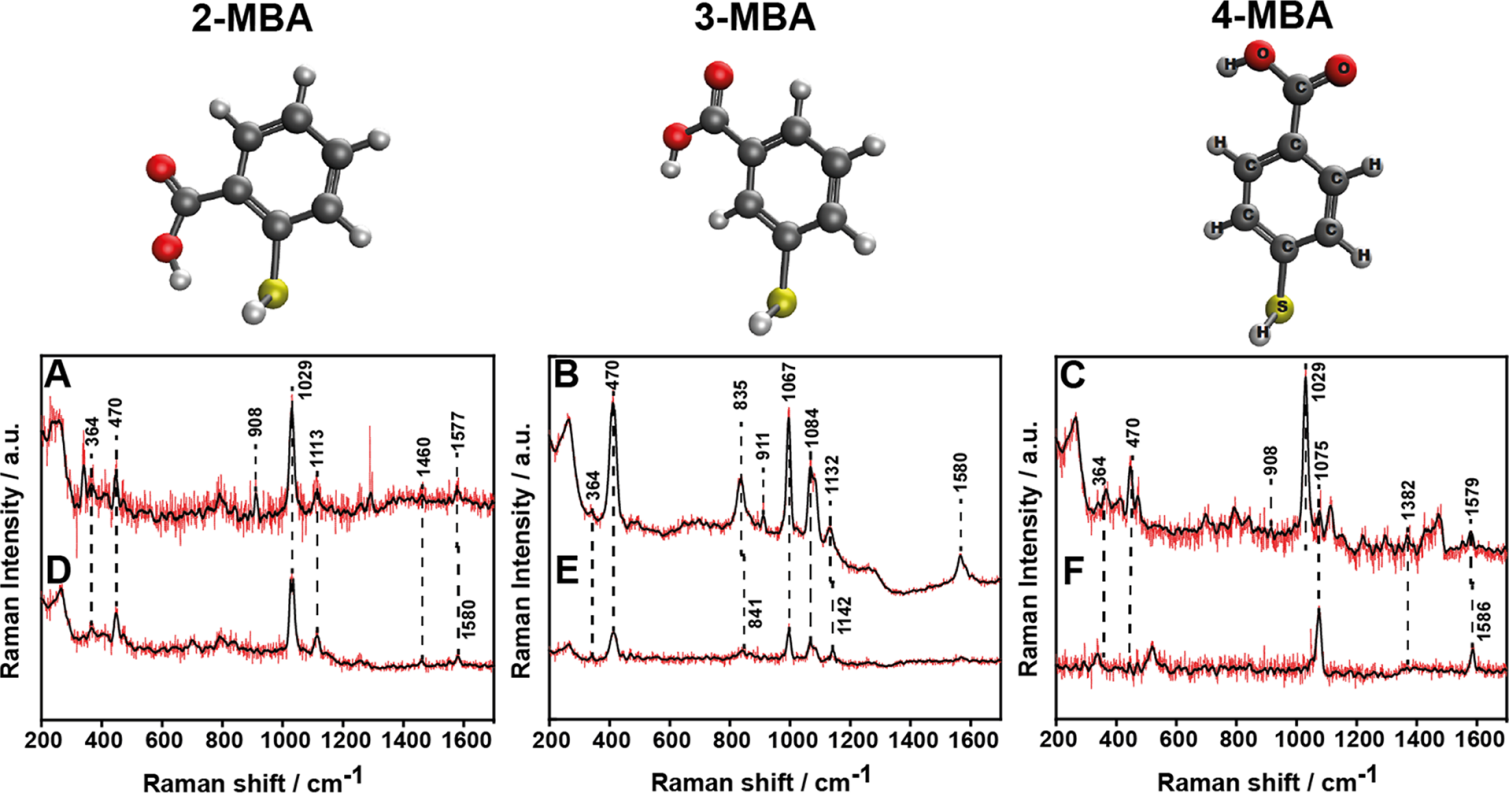
SERS spectra of AuNPs stabilized with different isomers of MBA. A, B, and C with and D, E, and F without an ADS matrix. The spectra were normalized to their maximum intensity. Raman experiments were conducted at 785 nm with a 300 mW intensity.

The adsorption of MBA isomers on an Au surface has been reported by Crooks^48^ who showed that 4-MBA creates a self-assembled-like monolayer on Au, whereas 2- and 3-MBA form inter-ligand interactions on the Au surface, which results in a less dense monolayer than 4-MBA. A similar behavior was reported on AuNPs.^49,50^ Interestingly, the difference in the assembly of the various MBA isomers on an identical AuNP is revealed in the electrochemical oxidation experiments (Figure S14). A shift of the AuNP oxidation peak potential can clearly be seen, which depends on the specific MBA isomer. Specifically, the peak potentials of the 2-, 3-, and 4-MBA are 0.88, 0.90, and 0.93 V, respectively. To the best of our knowledge, such shifts have never been reported and depend on both the capping agent as well as the matrix. Yet, this will be reported elsewhere. As discussed above, it is evident that the high selectivity observed by the NAIM systems cannot be attributed only to the size matching between the NPs and the nanovoids; the chemical interactions must play a major role as well. Therefore, we focused on understanding the NP–matrix interactions, using an additional surface-sensitive tool, that is, SERS.^24^ The details of the SERS experiments are described in the Experimental Section. We compared the SERS spectra of adsorbed AuNPs-MBA acquired with and without a diazonium-based matrix (Figure 5). The Raman band assignments of the AuNPs-MBA and AuNPs-MBA\ADS-COOH are listed in Tables S3–S5.

The most prominent features in the SERS spectra, which indicate a successful functionalization of the thiol groups on the AuNPs surface are the S–Au vibration at ∼264 cm^–1^ and C–S bending at ∼364 and 470 cm^–1.^^51,52^ These bands are detected in all six systems, namely, for every isomer-stabilized AuNPs with and without the matrix. It is well known that the S–H stretching at 2580 cm^–1^ is the most characteristic band of the thiol functional groups.^53^ The absence of this peak (not shown in our spectra) certifies that all the thiolate-based ligands are bound to the NPs through the sulfur group.^51,54^

The Raman intensities and the characteristic peaks of 2-MBA are strong and distinct at natural pH. Three bands can be correlated with the benzene ring; the band at 1113 cm^–1^ is assigned to the CH in-plane bending, that at 1580 cm^–1^ is assigned to the C=C symmetric stretching, and a band at 1460 cm^–1^ assigned to the ring bending mode. It is well documented that 2-MBA adsorbs on a metallic surface at neutral pH (in pure water) via both its sulfur and carboxylate groups and appears as a sulfobenzoate.^55^ Peaks associated with the carboxylic acid group are more challenging to analyze. For example, a lower frequency band of a C–COOH stretching mode at ∼790 cm^–1^ is apparent, while the COO^–^ symmetric stretching vibration at 1402 cm^–1^ is absent. The C–COOH symmetric band is red-shifted by 15 cm^–1^ from the benzoate literature peak. Such a red-shift is known for other carboxylic acids that adsorb on the surface through the carboxylate groups. Furthermore, the two intense bands, that is, the COO^–^ symmetric stretching at 1429 cm^–1^ and the COO^–^ bending at 806 cm^–1^, which are attributed to the benzoate are absent. The AuNPs for both ADS-covered and non-covered surfaces have bands at 558 and 552 cm^–1^, respectively, that are assigned to the Au–OH bond vibration (reported in the literature at 550–580 cm^–1^^56−58^). This means that the capping agent 2-MBA is connected to the AuNP through the thiolate and the carboxylate groups, yet we also detect the presence of COOH.

The SERS spectrum of AuNPs stabilized by 3-MBA is dominated by bands at 1142, 1084, and 841 cm^–1^, which are involved in in-plane CH deformation, a combination of C–S stretching and in-plane ring deformation, and COO^–^ bending, respectively. According to the selection rules, the appearance of the band at 1142 cm^–1^ indicates that the benzene ring of 3-MBA is not lying flat on the AuNP surface, unlike 2-MBA. Furthermore, the appearance of a low-frequency peak at 1668 cm^–1^ is associated with COO^–^ stretching rather than COOH. Another apparent peak is the 1067 cm^–1^ CH in-plane bending, which implies a certain angle between the plane of the benzene ring and the particle surface,^25,59^ thus alluding to a single connection of the thiol to the particle through the sulfur group.

The characteristic peaks of 4-MBA can be divided into two: those attributed to the carboxylic acid (1382 and 1669 cm^–1^) and those associated with the benzene ring (1075, 1460, and 1586 cm^–1^).^25,59,60^ The Raman intensity of the COO^–^ symmetric stretching at 1382 cm^–1^ is low, which indicates weak interaction between the carboxyl acid and the AuNP surface.^52^ This signal suggests that the distance between the carboxyl acid and the surface is large and 4-MBA generates a self-assembled-like monolayer on the AuNPs surface. Furthermore, the intensity of the band of the benzene ring stretching at 1587 cm^–1^ is stronger than most other bands, which indicates the existence of a certain angle between the plane of the benzene ring and the AuNP surface, similar to self-assembled monolayers. To conclude, our findings clearly confirm that the three isomers of MBA are organized differently on the AuNPs. The organization of 3-MBA on the AuNP is an intermediate between 2- and 4-MBA,^61^ namely, while the 2-MBA lies parallel to the surface and the 4-MBA is oriented toward the solution, 3-MBA is tilted.

The next step is to analyze the Raman spectra with the ADS matrices and to point out the changes appearing as a result of forming the matrix and the matrix effect on the different isomeric ligands. Figure 5 shows a clear amplification of the band intensities assigned to the benzene ring and the carboxylic acid of both the MBA and the matrix. This implies that the matrix must be near the NP surface. A similar behavior was observed for the 2- and 4-MBA, for example, the 1029 cm^–1^ band, which corresponds to the matrix ring breathing appeared for both, whereas it is undetectable for the 3-MBA. Moreover, the peak at ∼1580 cm^–1^ was red-shifted in both cases. The peaks of the carboxylic acid (of the 2 and 4-MBA) at 1668 and 1693 cm^–1^ disappeared and remained unchanged, respectively. In the case of 3-MBA, the COO^–^ and CH bending peaks at 841 and 1142 cm^–1^ red-shifted to 835 and 1132 cm^–1^, respectively. Furthermore, a COO^–^ peak appeared at 1372 cm^–1^ for the 2- and 3-MBA NPs inside the matrix, implying that this band is presumably associated with the matrix rather than the MBA on the NP.

**Figure 6:**
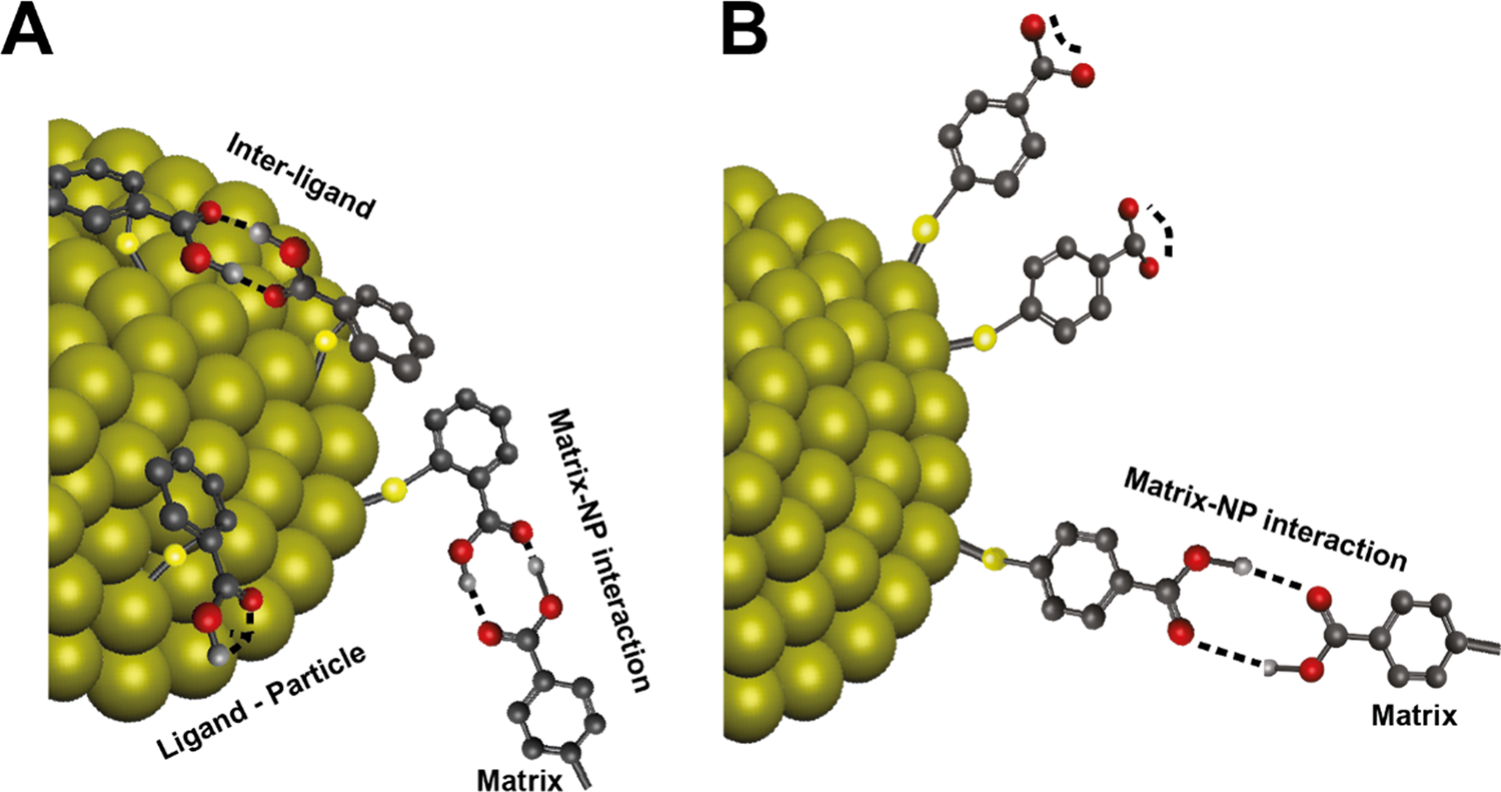
Schematic illustration of the NP–matrix interactions. (A) AuNPs-2-MBA showing inter-ligand, ligand–particle, and ligand–matrix interactions and (B) ligand–matrix interactions of the AuNPs-4-MBA.

All these changes in the Raman spectra allow us to draw some significant conclusions regarding the NP–matrix interactions. The disappearance of the COO^–^ band at 1668 cm^–1^ in the case of 2- and 4-MBA (upon forming the matrix) suggests that the carboxylate group was changed into a carboxylic acid due to binding through one of the oxygen atoms to either the AuNP or via hydrogen bonding to the matrix. We believe that 2-MBA is partially bound to the NP surface through the carboxylic acid, while the 4-MBA carboxylic acid is hydrogen bonded to the matrix (Figure 6). We recall that the carboxylic group on the 4-MBA does not bind to the metal surface.

Furthermore, the strong band at 908 cm^–1^ related to the 2- and 4-MBA and the band at 911 cm^–1^ of the 3-MBA in the respective matrices corroborate with the formation of a carboxylic acid dimer (homodimer). It is known that the homodimer frequency peak reported at 917 cm^–1^ of the in-phase vibration is red-shifted to 912 cm^–1^ as a consequence of the combination of in-phase and out-of-phase vibrations.^62^ These peaks are detected neither in the SERS spectra of NPs without the matrix nor in the electrografted ADS on a gold surface. Hence, this band can be attributed to the vibrational coupling of the carboxylic acid dimer created between the capping agents on the AuNPs and the functional matrix. As SERS amplifies the signals near the AuNPs, and the dimer peak amplitude is substantial in the AuNPs-ADS systems exclusively, we believe that this peak is a measure of the AuNP–matrix carboxylic acid dimer interaction.^63,64^

Hence, these results can shed light on the partial selectivity that is observed in the reuptaking experiments. The interchangeable reuptake between the AuNPs-4-MBA and AuNPs-2-MBA can be attributed to the capping agent–matrix intermolecular hydrogen bonding to form a dimer-type structure (Figure 6). This is presumably less preferable in the AuNPs-3-MBA where hydrogen bonding is more likely to prevail through interactions of the capping agent on the same NP. The AuNPs-3-MBA is, therefore, quite different from the two other isomers. Moreover, the structure of the 3-MBA is more bulky than the other isomers, which is likely to make a greater effect on the size and structure of the AuNPs-3-MBA. This can explain, at least qualitatively, the difference in its reuptake by the other systems. We are currently using DFT calculations to establish a more quantitative approach for this difference.

## Conclusions

This study is a substantial step toward understanding the imprinting of functionalized NPs in an organic matrix. We examined the recognition of nanovoids formed by imprinting AuNPs stabilized by the three isomers of MBA. We found a remarkable selectivity that must be attributed to chemical pairing, namely, to the specific interactions between the stabilizing isomer of the NP and the matrix. Therefore, we thoroughly investigated the NP–matrix interactions by different means and in particular by Raman spectroscopy. Significant shifts in the Raman bands for the AuNPs stabilized with the different MBA isomers were correlated with the shell–matrix interactions. Furthermore, distinct differences in the Raman spectra were also detected between the MBA stabilized AuNPs with and without the aryldiazonium-based matrix. Careful analysis of the Raman and electrochemical data enabled determining the orientation of the MBA adsorbed on the AuNPs as well as the nature of the hydrogen bonding, which was prominent in the NP–matrix interactions. We conclude that both the organization of the capping isomers on the AuNPs as well as the specific MBA–matrix interactions are responsible for the high reuptake selectivity observed for this system. Hence, this study shows that selective interactions between nanomaterials and a soft matrix can be fine-tuned by small changes in the capping agent. We believe that this concept can find interesting applications in various fields such as separation and sensing.

## Experimental Section

### Materials

Tetrabutylammonium tetrafluoroborate was obtained from ABCR (Karlsruhe, Germany). Ethanol (reagent grade) was ordered from J.T. Baker. Trisodium citrate (99%) was obtained from BDH. 4-Aminobenzoic acid (99%), tetrafluoroboric acid solution (HBF_4_, 50%), sodium nitrite (99%), chloroauric acid hydrate (HAuCl_4_·3H_2_O, 99.9%), potassium hexacyanoferrate(III), 2-MBA (99%), 3-MBA (99%), 4-MBA (99%), PEI aqueous solution (0.72 mg·mL^–1^, Mw = 800 g·mol^–1^), and sodium hydroxide were purchased from Sigma-Aldrich. Acetone (AR grade) was obtained from Gadot, Israel. Acetonitrile (ACN, gradient grade) was purchased from Bio-Lab. Hydrochloric acid (gradient grade) was obtained from Loba Chemie. All the chemicals were used as received. One side-coated ITO plates (7 mm × 50 mm × 0.7 mm) were purchased from Delta Technologies (CG-601 N-CUV, Stillwater, MN, USA). Dialysis tubing membrane (MWCO 12–14 kDa) was ordered from Medicell Membranes Ltd. (Liverpool, London). Ultrapure deionized water (Easy Pure UV, Barnstead) was used for all aqueous solutions.

### Instruments

CV and LSV were conducted with a CHI-630 (CH Instruments Inc., Austin, TX) potentiostat using a three-electrode setup glass cell. An Ag/AgCl (KCl 1 M) and Ag/AgBr quasi-reference electrodes were used for the aqueous and organic solution, respectively. A Pt wire was used as the counter electrode. Extra-high-resolution SEM (Magellan XHR 400 L, FEI), high-resolution TEM (Tecnai F20 G2), and FIB (460F1 Dual Bean, FEI Helios Nano Lab) were used to characterize the NAIMs. ζ-Potential was measured by dynamic light scattering (Zetasizer, Malvern ZS). The conductivity of the dialysis bath was measured by an Exstik EC400 conductometer (EXTECH Instruments).

#### Raman Measurements

Raman measurements were conducted using the InVia Confocal Raman Microscope (Renishaw) equipped with a 785 nm laser with a 300 mW intensity. The samples were exposed to 10% laser intensity for 50 s. The laser power density, the accumulation time, and the number of repetitions were varied to obtain an appropriate signal-to-noise ratio.

The measurements were conducted with ITO plates coated with AuNPs stabilized with different capping agents and the ADS-COOH as a matrix exactly according to the reported experimental conditions. The measurement of the ADS-COOH powder was exposed to 0.1% laser intensity for 10 s with the same laser wavelength and intensity. All the samples were accumulated 5 times to obtain an appropriate signal-to-noise ratio.

### Procedures

#### Synthesis of AuNPs

AuNPs (∼10 nm diameter) stabilized with citrate (AuNPs-cit) were synthesized based on the Turkevich method^26^ with some minor changes. Specifically, 97 mg of trisodium citrate was dissolved in 150 mL of water (2.2 mM) and heated to boiling under vigorous stirring. Then, 1 mL of an aqueous solution of 25 mM HAuCl_4_ 3H_2_O was added and stirred for 10 min until a red color was obtained.

##### Ligand Exchange of AuNPs by Structural Isomers of MBA

The AuNPs-cit were ligand-exchanged by 2, 3, and 4-MBA. The pH of the aqueous solutions of the thiols was changed from 2 to 6 by adding small amounts of 0.1 M NaOH. Then, 1 mL of the thiol at pH 6 was added into 11 mL of the seed solution and diluted with 22 mL of water. The mixture was mildly shaken (90 strokes min^–1^) for 24 h. The color of the solution did not change throughout the whole procedure. These AuNP MBA solutions were transferred to the dialysis tubing membrane for the removal of excess ligands. The reaction was initiated by dialyzing against ultrapure deionized water at room temperature, with continuous stirring for 24 h. The volume of the ultrapure deionized water in the dialysis chamber was always fixed at 2 L. To monitor the dialysis progress, the conductivity of the ultrapure deionized water was measured once every hour.

##### Preparation of ADS-COOH Tetrafluoroborate

4-Aminobenzoic acid (4-MBA, 5.00 g, 36.5 mmol), 20 mL of water, and HBF_4_ 48% (5.0 mL, 38.5 mmol) were added into a 100 mL flask equipped with a magnetic stirrer and an efficient condenser cooled with tap water. The mixture was heated gently for 10 min and was cooled to 0 °C with a water-ice bath. A cold solution of sodium nitrite (2.55 g, 37 mmol in 20 mL water) was added at 0 °C in 4 portions over 2 min. The yellow suspension was stirred for 20 min, filtrated, washed with 10 mL of 10% NaBF_4_ solution and 10 mL of water, and lyophilized for 1 h. The pale-yellow powder (ADS-COOH) was kept at −4 °C.

#### Imprinting Experiment

In a typical imprinting experiment, ITO plates were cleaned by sonication in acetone, ethanol, and twice with deionized water for 10 min. Then, the ITO plates were immersed in a PEI aqueous solution (0.72 mg·mL^–1^) for 2 h with mild shaking (100 strokes/min). The plates were washed for 24 h by mild shaking (100 strokes/min) with deionized water. This treatment avoided the excess of PEI chains that were not completely adsorbed to the ITO. Then the ITO/PEI surfaces were placed vertically in a solution of AuNPs stabilized with different structural isomers of MBA ligand for 1 h. Then, the plates were dried with a flow of Ar for 1 min. The samples were immersed in 5 mM of ADS solution. Electrografting was carried out by cycling the ITO/PEI/AuNPs between 0.5 and −1 V with a scan rate of 0.1 V s^–1^. The samples were washed with ACN and dried again with Ar for 1 min. The removal of the imprinted AuNPs from the matrix was achieved by electro-oxidation using a three-electrode cell in a 0.1 M HCl solution. A few LSV scans from 0.2 to 1.3 V at 0.1 V s^–1^ were performed until no oxidation peak was observed, indicating the complete removal of the AuNPs. The reuptake of the AuNPs was carried out by immersing the oxidized samples in a solution of AuNPs for 1 h, followed by careful washing with water and drying with Ar. Then, LSV was performed (same conditions as before) for measuring the amount of AuNPs, which was reabsorbed into the imprinted voids. The area of the samples electrochemically oxidized was always 12 mm × 7 mm.

#### Oxidation Peak Calculation

The LSV peak areas were calculated using an automatic method with MATLAB. This program written by us is used to determine the area under the LSV curve precisely and repetitively while being efficient. As numerous oxidation curves were analyzed, automating the process of curve integration was useful and time-saving.

#### Computational Details

DFT Calculations were carried out with the Q-Chem software using the B3LYP method and 6-311++G** basis set to obtain the vibrational RAMAN frequencies of the ADS-COOH. Initially, the geometry was optimized to the minimum of the potential energy surface. The employed basis set (6-311++G**) is widely preferred to obtain vibrational parameters.^65^ The spectrum was then compared to the obtained experimental data, and deviations of up to 50 cm^–1^ were found, which is consistent with the literature.^66^

## Supporting Information Available

Additional experimental details, including microscopy images, spectroscopy and electrochemistry data and SERS tables with all acquired peaks, and description of the vibration frequencies video (PDF)
