# Supplementary material for: High Recognition of Isomer-Stabilized Gold Nanoparticles through Matrix Imprinting
Source: ACS Appl Mater Interfaces. 2023 Jun 26;15(27):32687–96. doi: 10.1021/acsami.3c04311 (PMC10347121; doi:10.1021/acsami.3c04311)
Supplement: Supplementary file 1 — am3c04311_si_001.pdf [file am3c04311_si_001.pdf]

Supporting Information

High Recognition of Isomer Stabilized Gold Nanoparticles through Matrix Imprinting

Din Zelikovich[+]**, Pavel Savchenko**[+]**, Daniel Mandler***

Institute of Chemistry, The Hebrew University of Jerusalem, Jerusalem 9190401, Israel

Daniel.mandler@mail.huji.ac.il

[+]These authors contributed equally.

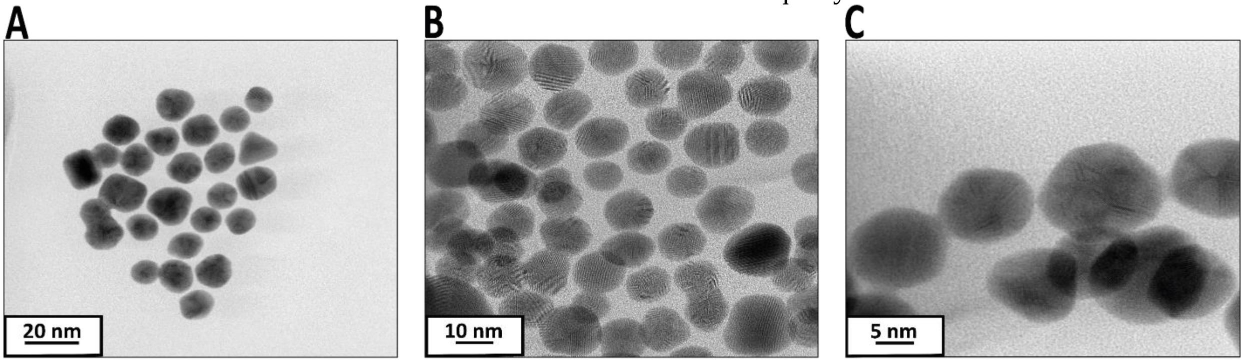

Figure S1: High-resolution TEM images of AuNPs stabilized by 2-MBA in different magnifications. (A) 20nm, (B) 10nm and (C) 5nm.

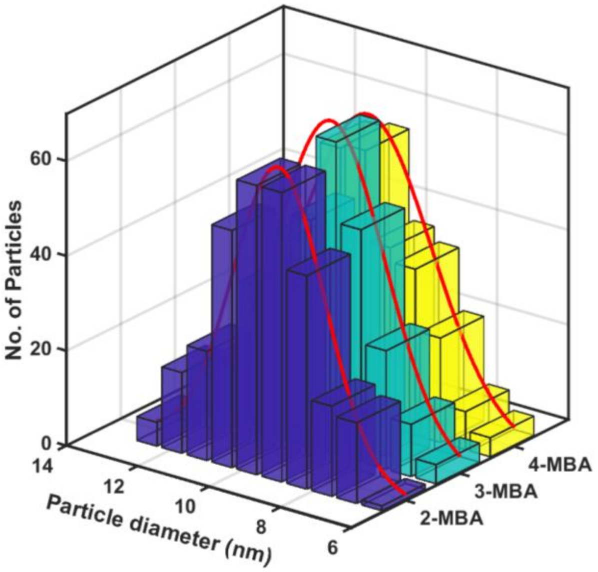

Figure S2: Size distribution histogram and Gaussian fitting of AuNPs stabilized by different ligands obtained from measuring 300 nanoparticles for each sample. The average size of 2-MBA, 3-MBA and 4-MBA are 9.5 ± 1.2, 9.6 ± 1.5 and 10.1 ± 1.6 nm, respectively.

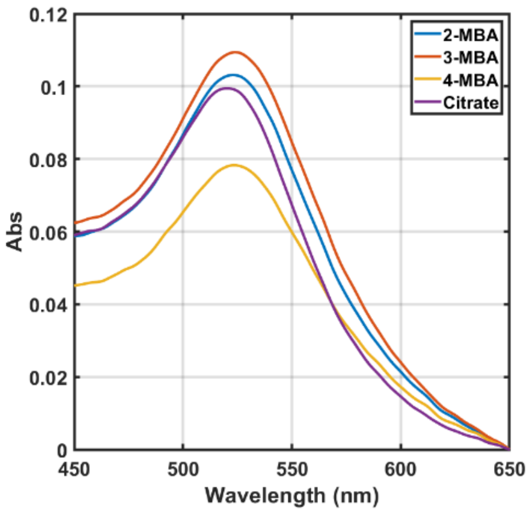

Figure S3: UV−visible spectra of the AuNPs stabilized by citrate and MBA isomers. λ_{max} for citrate, 2-MBA, 3-MBA, and 4-MBA are 521, 523, 524, and 523 nm, respectively.

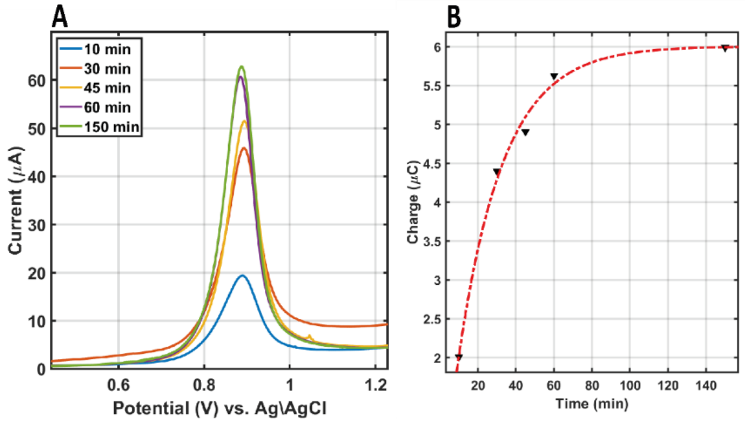

Figure S4: Time-dependent study of AuNPs-2MBA adsorption on ITO/PEI electrode. (A) LSV of AuNPs-2MBA after different adsorption times. (B) The charge due to oxidation of AuNPs adsorbed on the ITO surface as a function of time of adsorption.

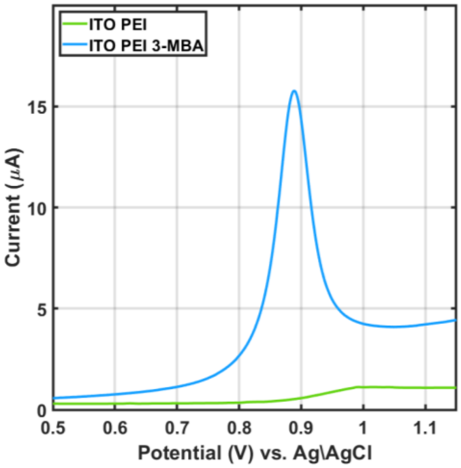

Figure S5: LSV of ITO/PEI surface in 0.1 M HCl solution. Shown are the oxidation waves of the treated ITO surface with (blue) and without (green) AuNPs-3-MBA.

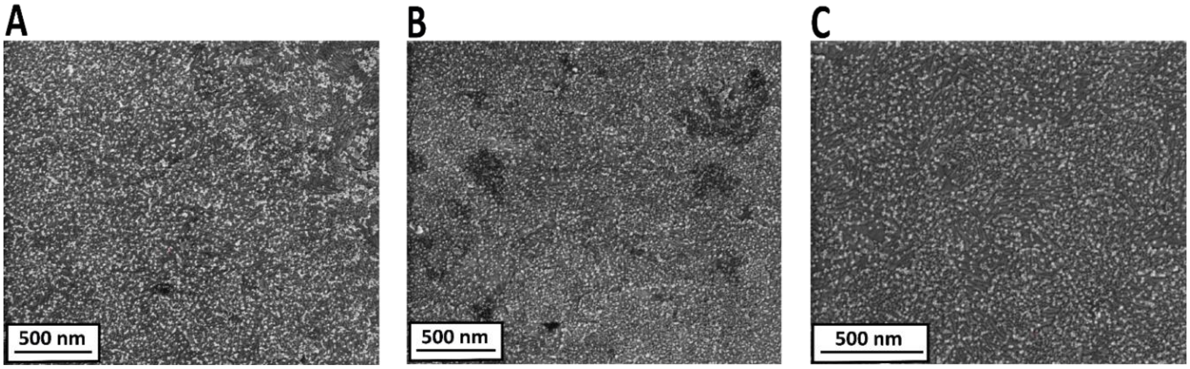

Figure S6: SEM images of ITO surfaces treated with PEI solution (0.72 $mg\ ml^{-1}$) for 1 h followed by adsorption of AuNPs stabilized by: (A) 2-MBA, (B) 3-MBA, and (C) 4-MBA.

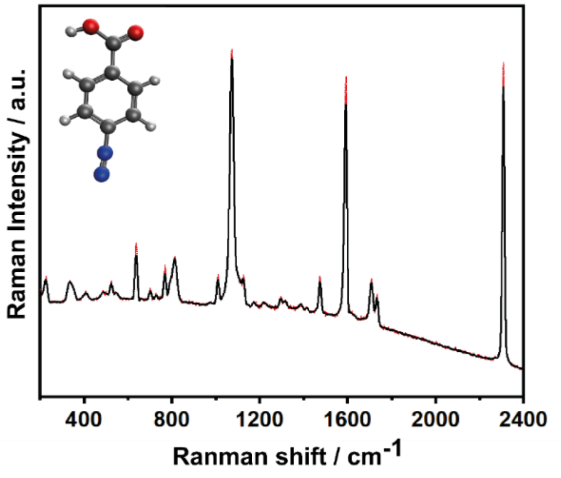

Figure S7: Raman spectrum of ADS-COOH. The spectrum was normalized to its maximum intensity. Raman experiment was conducted at 785 nm with a 300 mW intensity. The ADS-COOH molecule shown in the inset is plotted in its optimized geometry.

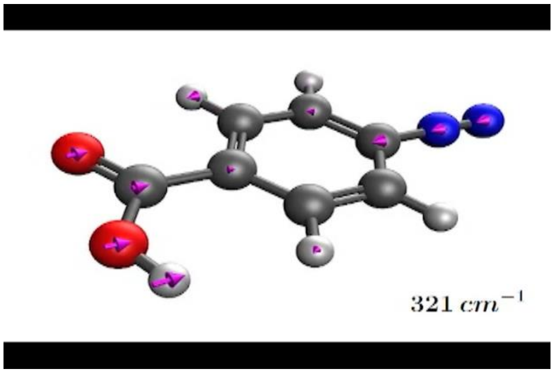

Figure S8: A video of the most significant vibration of the ADS-COOH acquired from DFT calculations.

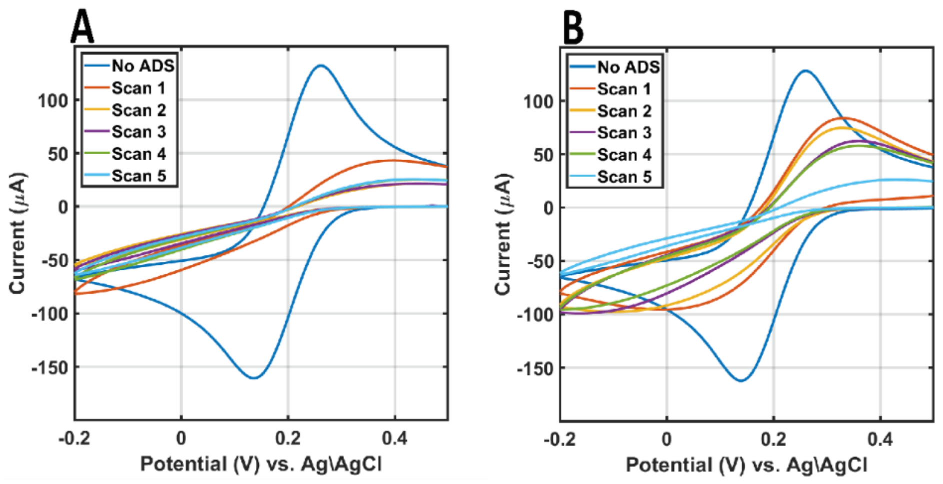

Figure S9: CV of ITO/PEI electrodes in 2 mM $K_3[Fe(CN)_6]$ and 0.1 M KCl: (A) without and (B) with AuNPs-3-MBA. The scan rate was 0.05 V s^{-1}.

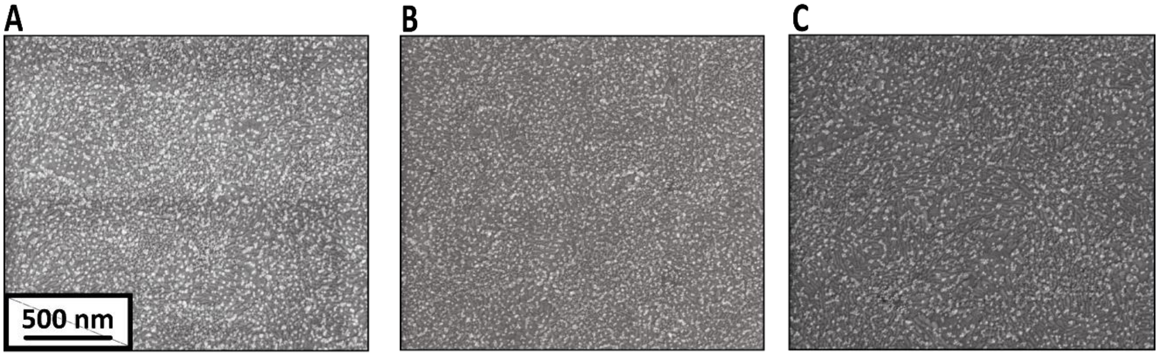

Figure S10 SEM images of ITO/PEI after adsorption of: (A) 2-MBA, (B) 3-MBA and (C) 4-MBA and electrografting of ADS-COOH (3 scans).

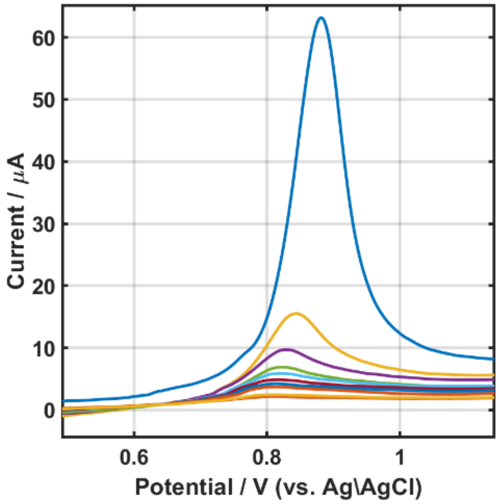

Figure S11 10 consecutive LSV oxidation curves of ITO/PEI/AuNPs-3-MBA/ADS-COOH in 0.1 M HCl solution (scan rate 0.1 V s^{-1}).

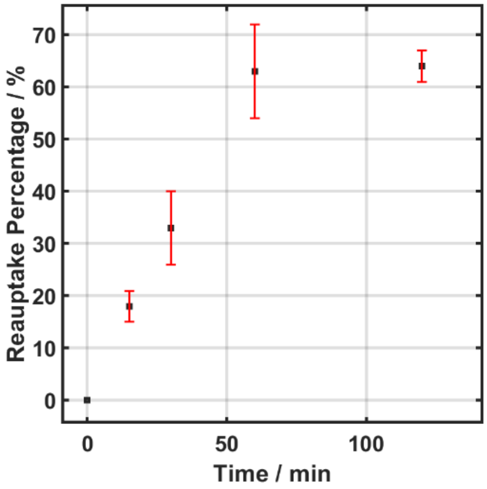

Figure S12: Time-dependent reuptake of AuNPs-3-MBA from their solution.

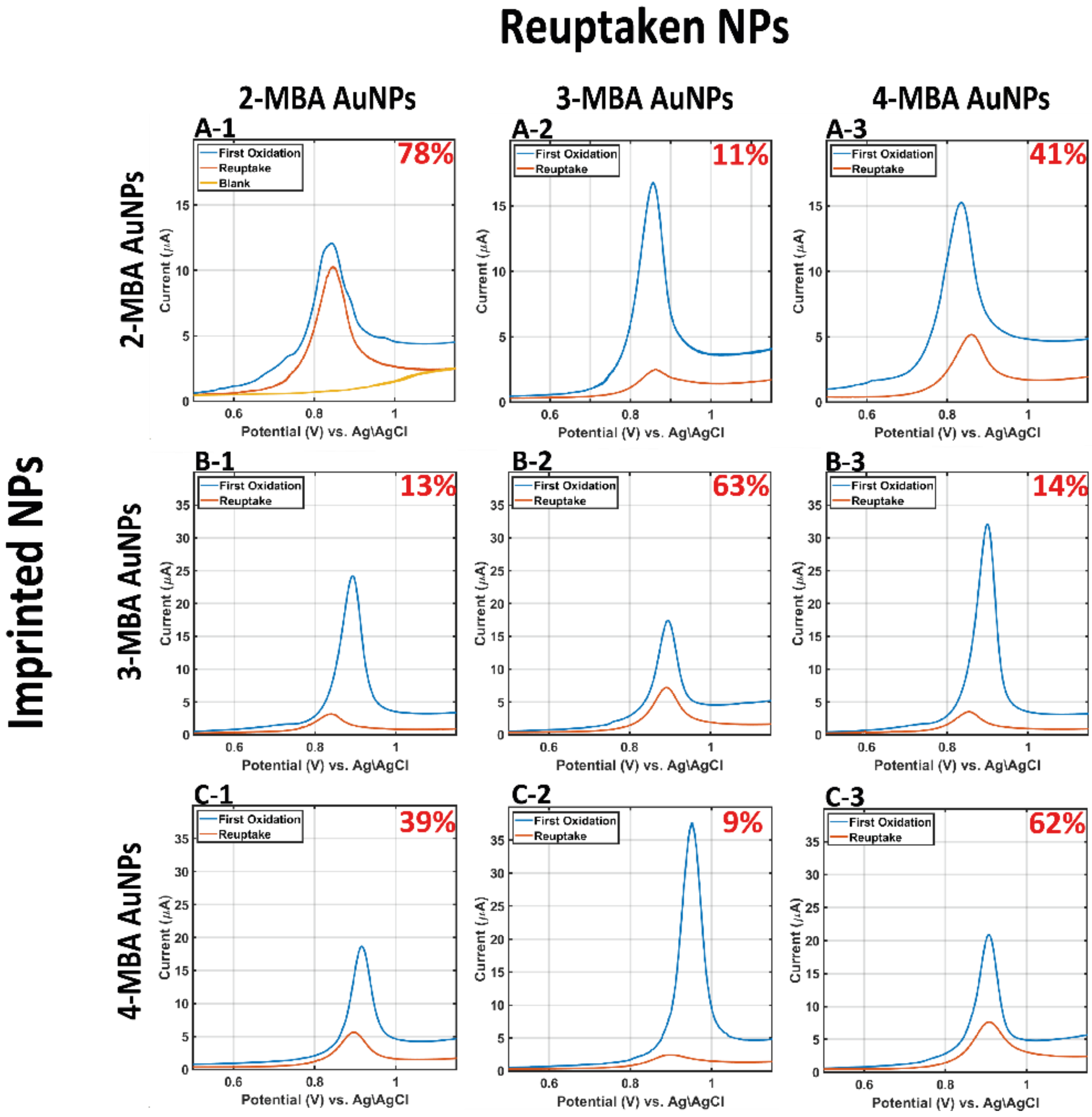

Figure S13: LSV of ITO/PEI/ADS-COOH in 0.1 M HCl: A1-3 are samples imprinted with AuNPs-2-MBA and immersed in AuNPs-2-MBA, AuNPs-3-MBA and AuNPs-4-MBA solutions, respectively. B1-3 are samples imprinted with AuNPs-3-MBA and immersed in AuNPs-2-MBA, AuNPs-3-MBA and AuNPs-4-MBA solutions, respectively. Finally, C1-3 are samples imprinted with AuNP-4-MBA and immersed in AuNPs-2-MBA, AuNPs-3-MBA and AuNPs-4-MBA solutions, respectively. The yellow curve is LSV of a non-imprinted film. Scan rate was 0.1 V s^{-1}.

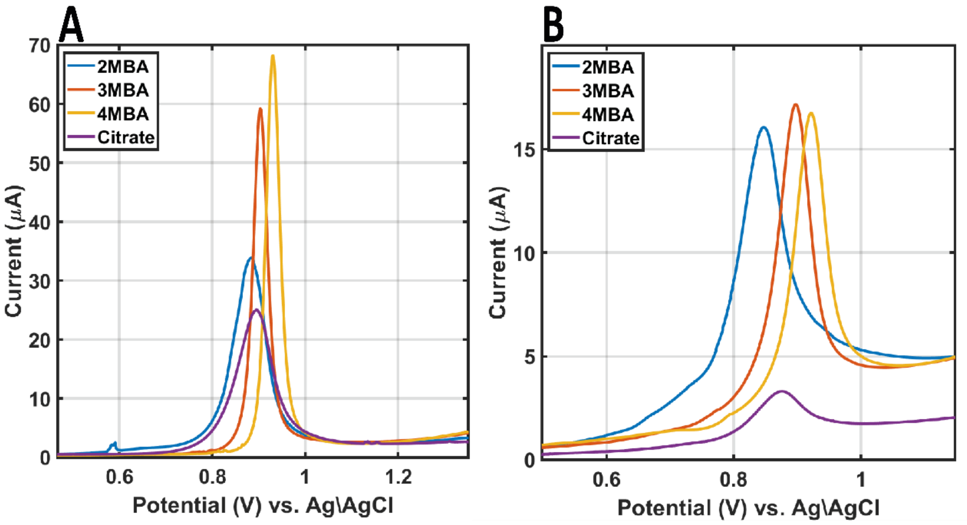

Figure S14: LSV oxidation curves of ITO/PEI/AuNPs-MBA in 0.1 M HCl solution. (A) without and (B) with ADS-COOH matrix (scan rate 0.1 V s^{-1}).

	AuNPs-2-MBA	AuNPs-3-MBA	AuNPs-4-MBA	AuNPs-cit
ζ-potential/mV	-45.1±1.9	-42.4±1.3	-47.3±2.6	-51.2±2.0

Table S1: ζ-potential of the AuNPs stabilized by the different capping agents.

Raman peak ADS powder (cm^{-1})	Theoretical model DFT Raman peak (cm^{-1})	Assignment	Ref.
335	321	Ring deformation	
409	398	Out-of-plane deformation and twist transform of the aromatic ring	1-3
486	475	In-plane deformation (rocking) of the carboxylic group	
546	559	Out-of-plane deformation of the aromatic ring; boat transforms; C–N–N in-plane deformation (scissors)	2 ,1
636	644	Asymmetric ring stretch	2 ,1
728	727	Ring deformation	
768	740	CH out-of -plane ring wagging	
812	820	Ring breathing	1
1009	1023	Ring breathing	4
1073		CH in-plane bending for p– and mono–substituted benzenes	5 ,4
1124	1148	$C–N_2$ stretch	4 ,2
1590	1583<br>1594	Asymmetric C=C stretch in the ring<br>“Quinoid” vibration	2 ,1
1707<br>1732	1669	C=O stretching	5
2308	2258	N=N stretching	5 ,2 ,1
3099	3215	CH in-plane ring wagging	5 ,1

Table S2: Experimental and calculated Raman bands of ADS-COOH.

2-MBA (cm^{-1})	2-MBA with ADS (cm^{-1})	Assignment	Ref.
264	257	S–Au Covalent binding	6
-	340	Ring Deformation	
364	364	C–S bending vibration	7
471	471	C–S bending vibration	7
552	558	Au-OH	8-10
790	789	C–COOH stretching	11, 12
-	908		
1031	1029	Ring breathing	4
1113	1113	Ring stretch and CH in-plane	12 ,11
-	1372	COO^{-} stretching	14 ,13
1460	1460	CH in-plane ring bending	15 ,12
1580	1577	C=C stretching	15 ,12 ,11
1668	1669	COO^{-} stretching	
1693	1695	C=O stretching vibration	16

Table S3: Wavenumbers and band assignments of the SERS bands of 2-MBA with and witout the matrix

3-MBA (cm^{-1})	3-MBA with ADS (cm^{-1})	Assignment	Ref.
264	264	S–Au Covalent binding	6
340	340	Ring Deformation	
411	411	Out-of-plane deformation and twist transform of the aromatic ring of the ADS-COOH	2 ,1
470	470	C–S bending vibration	7
-	648	Asymmetric ring stretch	5 ,3
841	835	COO^{-} bending	17
-	911		
1067	1067	CH in-plane bending	13
1084	1084	Combination of C-S stretching and in-plane ring deformation	17 ,13
1142	1132	In-plane CH deformation	17 ,13
-	1369	COO^{-} stretching	14 ,13
1570	1570	C-C stretching	17
1668	1670	COO^{-} stretching	16

Table S4: Wavenumbers and band assignments of the SERS bands of 3-MBA with and witout the matrix

4-MBA (cm^{-1})	4-MBA with ADS (cm^{-1})	Assignment	Ref.
264	264	S–Au Covalent binding	6
336	340	Ring Deformation	
364	364	C–S bending vibration	7
-	408	Out-of-plane deformation and twist transform of the aromatic ring	2 ,1
470	471	C–S bending vibration	7
518	-		18
842	842	COO^{-} bending	13
-	908		
-	1113	Ring stretch and CH in-plane	12 ,11
-	1029	Ring breathing of the ADS-COOH	4
1075	1076	CH in-plane bending	16 ,14 ,13 ,11
1382	1380	COO^{-} symmetric stretching	14 ,13
1460	1471	CH in-plane bending	16 ,14 ,11
1586	1579	Ring stretching	16 ,14 ,13 ,11
1669	-	COO^{-} stretching	14
1693	1694	C=O stretching vibration	16

Table S5: Wavenumbers and band assignments of the SERS bands of 4-MBA with and witout the matrix

1. Badawi, H. M.; Förner, W.; Ali, S. A., A Comparative Study of the Infrared and Raman Spectra of Aniline and o-, m-, p-Phenylenediamine Isomers. *Spectrochimica Acta Part A: Molecular and Biomolecular Spectroscopy* **2013,** *112*, 388-396.
2. Minaev, B. F.; Bondarchuk, S. V.; Gîrţu, M. A., DFT Study of Electronic Properties, Structure and Spectra of Aryl Diazonium Cations. *Journal of Molecular Structure: THEOCHEM* **2009,** *904* (1), 14-20.
3. Minaev, B. F.; Bondarchuk, S. V.; Gîrţu, M. A., DFT Study of Electronic Properties, Structure and Spectra of Aryl Diazonium Cations. *Journal of Molecular Structure: THEOCHEM* **2009,** *904* (1-3), 14-20.
4. Laurentius, L.; Stoyanov, S. R.; Gusarov, S.; Kovalenko, A.; Du, R.; Lopinski, G. P.; McDermott, M. T., Diazonium-Derived Aryl Films on Gold Nanoparticles: Evidence for a Carbon–Gold Covalent Bond. *ACS Nano* **2011,** *5* (5), 4219-4227.
5. Infrared and Raman Characteristic Group Frequencies: Tables and Charts. 3rd ed By George Socrates (The University of West London, Middlesex, U.K.). J. Wiley and Sons: Chichester. 2001. xviii + 348 pp. $185.00. ISBN: 0-471-85298-8. *Journal of the American Chemical Society* **2002,** *124* (8), 1830-1830.
6. Capocefalo, A.; Mammucari, D.; Brasili, F.; Fasolato, C.; Bordi, F.; Postorino, P.; Domenici, F., Exploring the Potentiality of a SERS-Active pH Nano-Biosensor. *Frontiers in Chemistry* **2019,** *7*.
7. Hong, S.; Li, X., Optimal Size of Gold Nanoparticles for Surface-Enhanced Raman Spectroscopy Under Different Conditions. *Journal of Nanomaterials* **2013,** *2013*, 790323.
8. Niaura, G.; Gaigalas, A. K.; Vilker, V. L., Surface-Enhanced Raman Spectroscopy of Phosphate Anions: Adsorption on Silver, Gold, and Copper Electrodes. *The Journal of Physical Chemistry B* **1997,** *101* (45), 9250-9262.
9. Murphy, P. J.; LaGrange, M. S., Raman Spectroscopy of Gold Chloro-Hydroxy Speciation in Fluids at Ambient Temperature and Pressure: A Re-Evaluation of the Effects of pH and Chloride Concentration. *Geochimica et Cosmochimica Acta* **1998,** *62* (21), 3515-3526.
10. Peck, J. A.; Tait, C. D.; Swanson, B. I.; Brown, G. E., Speciation of Aqueous Gold(III) Chlorides From Ultraviolet/Visible Absorption and Raman/Resonance Raman Spectroscopies. *Geochimica et Cosmochimica Acta* **1991,** *55* (3), 671-676.
11. Hong, Y.; Wang, R.; Jiang, Z.; Cong, Z.; Song, H., Rapid SERS Detection of Thiol-Containing Natural Products in Culturing Complex. *International Journal of Analytical Chemistry* **2020,** *2020*, 9271236.
12. Ma, C.; Harris, J. M., Surface-Enhanced Raman Spectroscopy Investigation of the Potential-Dependent Acid−Base Chemistry of Silver-Immobilized 2-Mercaptobenzoic Acid. *Langmuir* **2011,** *27* (7), 3527-3533.
13. Ma, W.-q.; Fang, Y.; Hao, G.-l.; Wang, W.-g., Adsorption Behaviors of 4-Mercaptobenzoic Acid on Silver and Gold Films. *Chinese Journal of Chemical Physics* **2010,** *23* (6), 659-663.
14. Liu, Y.; Yuan, H.; Fales, A. M.; Vo-Dinh, T., pH-Sensing Nanostar Probe Using Surface-Enhanced Raman Scattering (SERS): Theoretical and Experimental Studies. *Journal of Raman Spectroscopy* **2013,** *44* (7), 980-986.
15. Aswathy, B.; Sony, G.; Gopchandran, K. G., Shell Thickness-Dependent Plasmon Coupling and Creation of SERS Hot Spots in Au@Ag Core-Shell Nanostructures. *Plasmonics* **2014,** *9* (6), 1323-1331.
16. Jamieson, L. E.; Jaworska, A.; Jiang, J.; Baranska, M.; Harrison, D. J.; Campbell, C. J., Simultaneous Intracellular Redox Potential and pH Measurements in Live Cells Using SERS Nanosensors. *Analyst* **2015,** *140* (7), 2330-2335.
17. Velleman, L.; Bruneel, J.-L.; Guillaume, F.; Losic, D.; Shapter, J. G., Raman Spectroscopy Probing of Self-Assembled Monolayers Inside the Pores of Gold Nanotube Membranes. *Physical Chemistry Chemical Physics* **2011,** *13* (43), 19587-19593.
18. Wei, H.; Willner, M. R.; Marr, L. C.; Vikesland, P. J., Highly Stable SERS pH Nanoprobes Produced by Co-Solvent Controlled AuNP Aggregation. *Analy141* (17), 5159-5169.
